# Subcellular localization of APMCF1 and its biological significance of expression pattern in normal and malignant human tissues

**DOI:** 10.1186/1756-9966-28-111

**Published:** 2009-08-09

**Authors:** Yaqing Zhang, Qinlong Li, Feng Zhu, Jihong Cui, Kainan Li, Qing Li, Ruian Wang, Wenyong Wang, Weihua Wang, Wei Yan

**Affiliations:** 1 Department of Pathology, State Key Laboratory of GI Cancer Biology, Xijing Hospital, Fourth Military Medical University, Xi'an, 710032, Shaanxi Province, PR China; 2 Department of Environmental and Molecular Toxicology, North Carolina State University, Raleigh, NC 27695-7633, USA; 3 Department of Oncology, Jinan Military General Hospital, Jinan, Shandong, 250031, PR China; 4 Biotechnology Centre, School of Pharmacy, Fourth Military Medical University, Xi'an, 710032, Shaanxi Province, PR China

## Abstract

**Background:**

APMCF1 is a novel human gene first cloned from apoptotic MCF-7 cells. Our previous study found ectogenic APMCF1 could induce G1 arrest in hepatocarcinoma cell line HHCC. In order to search its broad expression profile for further understanding of its mechanism in tumor, we investigated a subcellular location of APMCF1 and performed an immunohistochemistry study including various tumor and normal tissues. Discovery from the expression characterization of AMPCF1 may have applicability in the analysis of its biological function in tumor.

**Methods:**

We investigated subcellular localization of APMCF1 by transient transfection in green monkey kidney epithelial cells (COS-7) with a fusion protein vector pEGFP-APMCF1 and detected expression profile in a broad range of normal and malignant human tissues via tissue microarray (TMA) by immunohistochemistry with polyclonal antibody first produced in our laboratory.

**Results:**

EGFP-APMCF1 was generally localized in the cytoplasm of COS-7 cell. Positive staining of APMCF1 was found in liver, lung, breast, colon, stomach, esophagus and testis, exhibited a ubiquitous expression pattern while its expression was up-regulated in tumor tissues compared with corresponding normal tissues. Normal brain neuron cells also showed expression of APMCF1, but negative in gliocyte cells and glioma. Both the normal and tumor tissues of ovary were absent of APMCF1 expression. Positive immunostaining for APMCF1 with large samples in liver, colon, esophagus, lung and breast carcinomas were 96% (51/53), 80% (44/55), 57% (30/53), 58% (33/57) and 34% (16/47) respectively.

**Conclusion:**

These results revealed a cytoplastic expression pattern of APMCF1 and up-regulated in tumour tissues suggesting APMCF1 may have potential relationship with oncogenesis. The data presented should serve as a useful reference for further studies of APMCF1 functions in tumorigenesis and might provide a potential anti-tumor target.

## Background

The APMCF1 gene was first isolated from the cDNA bank of breast carcinoma cell line MCF-7 cells treated with all-trans retinoic acid (ATRA) by an improved PCR-based subtractive hybridization strategy [1,2]. The cDNA is 1,745 bp in full length and is located in chromosome 3q23–24. The predicted protein of human APMCF1 contains a small GTP-protein (G protein) domain which suggests that APMCF1 is a novel member of the small G-protein superfamily [3,4]. More interesting is that APMCF1 and rat homolog named as signal recognition particle receptor β (SRβ) are of 271 and 269 amino acids, respectively, and are highly homologous (89% amino acid identity). Further analysis shows it also shares significant homology to the SRβ proteins of species such as Saccharomyces, C. elegan, Drosophila, and indicates that APMCF1 is human SRβ, a member of small G protein regulating intracellular vesicle trafficking, as well as a well-conserved protein [3-5].

Moreover, as a potential small G-protein, APMCF1 may play a key role in diverse cellular and developmental events like other identified small G-protein family members (i.e. the Ras and Rho), including differentiation, cell division, vesicle transport, nuclear assembly, and control of the cytoskeleton [6].

Currently, few literatures about the function study of this gene have been reported, especially in tumor. In order to learn more about the expression pattern and potential biological function of APMCF1 in other tumors, we detected APMCF1 subcellular localization and expression profile in a broad range of normal and malignant human tissues in this study.

## Methods

### Reagents

pGEM-APMCF1 and pEGFP-C1 have been characterized [3]. Restriction enzymes Hind-Ø, Sal I polymerase were purchased from Takara (Dalian, China). DMEM medium and FBS were obtained from Gibco-BRL (Gaithersburg, MD, USA). 1 kb Plus ladder, G418 and lipofectmin2000 were purchased from Invitrogen (Carlsbad, CA, USA).

### Samples

Six TMAs with one containing nine kinds of important human organs including their malignant tumor, tumor-adjacent tissues and normal tissues, and the others containing five kinds of frequent human epithelia carcinoma were involved in this study (Cybrdi Inc., Shaanxi, China). Table 1 and 2 listed detailed information of the tissues presented on the slides.

**Table 1:** Expression of APMCF1 in normal and malignant human tissues

Tissue type	Sample size	Score
Liver		
carcinoma tissues	2	+++/+++
tumor-adjacent tissues	2	++/++
normal tissues	2	++/+
Lung		
carcinoma tissues	2	+++/+++
tumor-adjacent tissues	2	+/+
normal tissues	2	+/+
Breast		
carcinoma tissues	2	++/+++
tumor-adjacent tissues	2	++/+
normal tissues	2	+/-
Stomach		
carcinoma tissues	2	++/++
tumor-adjacent tissues	2	+/-
normal tissues	2	-/-
Colon		
carcinoma tissues	2	+++/+++
tumor-adjacent tissues	2	+/+
normal tissues	2	++/-
Ovary		
carcinoma tissues	2	-/-
tumor-adjacent tissues	2	-/-
normal tissues	2	-/-
Esophagus		
carcinoma tissues	2	+++/+++
tumor-adjacent tissues	2	++/+++
normal tissues	2	+/+
Brain		
glioma tissues	2	-/-
tumor-adjacent tissues	2	+/-
normal tissues	2	+/+
Testis		
seminoma tissues	2	++/+
tumor-adjacent tissues	2	+/-
normal tissues	2	+/-

As indicated in the Methods section, APMCF1 immunolabeling was scored as follows: weak immunolabeling (+), moderate immunolabeling (++), strong immunolabeling (+++), and no immunolabeling (-).

**Table 2:** Expression of APMCF1 in human carcinomas

Tissue type	Sample size	Positive	Positive frequency (%)
Colon carcinoma	55	44	80
Esophageal carcinoma	53	30	57
Lung carcinoma	57	33	58
Hepatic carcinoma	53	51	96
Breast carcinoma	47	16	34

### Cell culture

Immortalized monkey kidney COS-7 cells were stocked in our lab. Cells were cultured in DMEM medium containing 10% fetal bovine serum, 50 IU/ml penicillin and 50 μg/ml gentamycin at 37°C under an atmosphere of 5% CO_2_.

### Plasmids

The entire APMCF1 coding region was amplified by PCR, using upstream and downstream primers which introduce a Hind III and Sal I site respectively according to the conjunct sequence. APMCF1 PCR primers were designed as follows: sense 5' ATAAGCTTCCATGGCTTCCG 3'; antisense 5' ACGCGTCGACCTGCCTCTCAGGCAAT 3'. pGEM-APMCF1 constructed by our lab previously [3] was used as templates for PCR amplification. PCR products were digested with Hind III and Sal I, and subcloned into pEGFP-C1, resulting in pEGFP-C1-APMCF1 to express APMCF1 protein fused to GFP. The recombinant plasmid was confirmed by Hind III and Sal I digestion and sequencing.

### Gene transfection

COS-7 cells which were seeded on glass cover-slips in 6 cm plates were cultured in DMEM medium containing 10% fetal bovine serum, and transiently transfected with the plasmid at 50–70% confluence using lipofectmin2000 reagent according to manufacturer instructions. Cover-slips were taken out of six-well plates after transfecting 24 h to use in the detection of expression with recombinant APMCF1 protein. As a control we used the pEGFP-C1 vector producing GFP protein.

### Immunohistochemical Analysis

Tissue sections on microscopic slides were processed through a graded series of alcohols and rehydrated in distilled water. Heat-induced antigen retrieval was performed by hydrated autoclaving in citrate buffer (10 mmol/L concentration, pH 6.0) for 5 min. To minimize non-specific background reactivity, tissue sections were incubated with normal goat serum for 10 min. The slides were cooled to room temperature for 30 min to complete antigen unmasking, and standard indirect biotin-avidin immunohistochemical analysis was performed to evaluate APMCF1 protein expression using a polyclonal anti-APMCF1 antibody (1:100 diluted) produced by our lab previously [3]. Incubation with non-immune rabbit serum and antibody blocked with purified APMCF1 protein served as a negative control.

Protein expression was scored by two observers as: absent (-); weakly positive (+), < 10% cells showed positive staining; moderately positive (++), 10–50% cells showed positive staining; or strongly positive (+++), > 50% cells showed positive staining.

## Results

### Subcellular localization of APMCF1 protein

**Figure 1:**
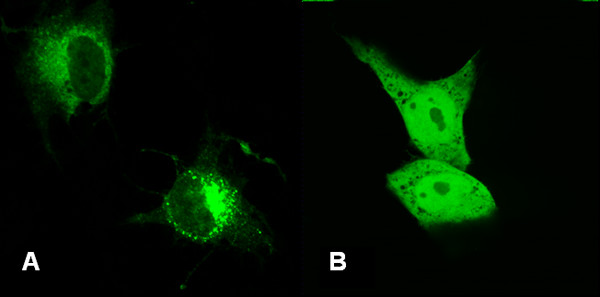
**Subcellular localization of the EGFP-APMCF1 fusion protein**. COS-7 cells were transfected with pEGFP-C1-APMCF1 or pEGFP-C1 vector. Twenty-four hours after transfection, subcellular localization of EGFP-APMCF1 fusion proteins was examined by direct fluorescent microscopy. (A) green fluorescence was seen in the cell cytoplasm of COS-7 cells transfected with pEGFP-C1-APMCF1; (B) green fluorescence was seen in the cell nuclei and cytoplasm of COS-7 cells transfected with pEGFP-C1.

For direct visualization of the cellular location of APMCF1, the corresponding cDNAs were cloned in frame with enhanced green fluorescent protein (EGFP) in the mammalian expression vector pEGFP-C1, followed by transient transfection into green monkey kidney epithelial cells (COS-7). Typical patterns are shown in Figure 1. In singly transfected cells, fluorescence was dispersed throughout the cytoplasm.

### Expression of APMCF1 in normal and malignant human tissues

Brown labeling represented the presence of APMCF1. The relative intensity was scored from (-) to (+++). Specific cytoplasmic staining was observed in the majority of positive stained cells, suggesting that APMCF1 was a cytoplasmic protein.

**Figure 2:**
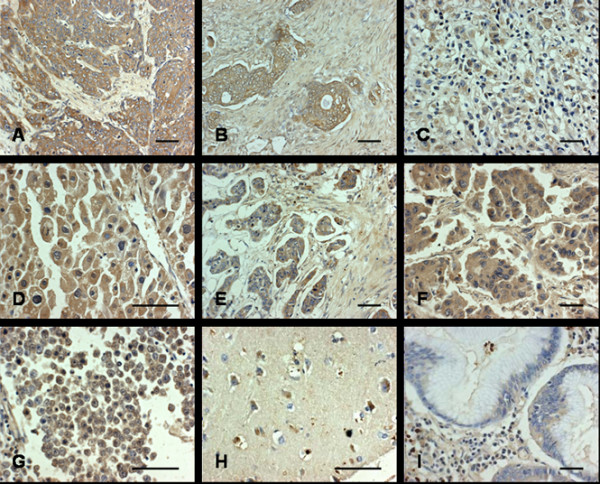
**Expression of APMCF1 in normal and malignant human tissues**. Expression of APMCF1 in normal and malignant human tissues was detected by immunohistochemistry. (A) esophagus carcinoma; (B) colon carcinoma; (C) gastric carcinoma; (D) liver carcinoma; (E) breast carcinoma; (F) lung carcinoma; (G) testis seminoma; (H) brain; (I) gastric mucosa. Bar = 50 μm.

Generally, APMCF1 was detected in the parenchymal cells of liver, lung, breast, colon, stomach, esophagus and testis, including the malignant tumor, tumor-adjacent tissues and normal tissues. Normal brain neuron cells also showed expression of APMCF1, but no detectable labeling was observed in brain gliocyte cells and glioma. Both the normal and tumor tissues of ovary were absent of APMCF1 expression. Representative photomicrographs are presented in Figure 2. A high expression level of APMCF1 was found in most of human carcinomas compared with that in their normal tissues (Table 1).

We also detect the specific expression pattern of APMCF1 in several common carcinomas including liver, colon, esophagus, lung and breast carcinomas in a large sample (Table 2). The positive ratios of APMCF1 in liver, colon, esophagus, lung and breast carcinomas were 96%, 80%, 57%, 58% and 34% respectively.

## Discussion

Small GTP-binding proteins (G proteins) are monomeric G proteins with GTPase structure in amino acid sequence structure and molecular masses of 20–40 kDa, currently existing in eukaryotes from yeast to human and containing more than 100 members. Based on both their sequence homology and function, they have been subdivided into at least six families: Ras, Rho, Rab, Sar1/Arf, Ran, and Rad/Gem [7,8]. They regulate a wide variety of cell functions in response to diverse stimuli, such as cell growth, apoptosis, lipid metabolism, cytoarchitecture, membrane trafficking, and transcriptional regulation [9-12]. However, uncontrolled activation of these multifunctional proteins (i.e. point mutations or overexpression) cause them insensitive to regulatory signals, leading to uncontrolled proliferation, enhanced angiogenesis, inhibition of apoptosis, and genetic instability, all of which result in tumor development [12-14]. Their cellular oncogenes were then identified, and their mutations were furthermore found in some human carcinomas [15-17].

The predicted protein of APMCF1 contained a GTPase domain closely related to ADP-ribosylation factor family (ARF) and Sar1p-like members of the Ras-family of small GTPases, suggesting it was a new member of small GTP-binding proteins and also a human homolog of SRβ [18]. The SR is a heterodimeric complex assembled by the two GTPases SRα and SRβ [5]. The eukaryotic signal recognition particle (SRP) and its receptor (SR) play a central role in co-translational targeting of secretory and membrane proteins to the endoplasmic reticulum (ER). In eukaryotes, this process is tightly controlled by the concerted action of three G proteins, the 54-kD subunit of SRP, SRα and SRβ [19-22]. All SRβ members in species other than human are cytoplasmic proteins. The subcellular location in present study based on APMCF1-GFP fusion protein identified that APMCF1 has a cytosol distribution pattern which also concoined it was a human homolog of SRβ.

There is little information about the function of AMPCF1 so far. As a human homolog of SRβ, APMCF1 might be involved in the targeting of nascent polypeptide chains to the protein translocation machinery in the endoplasmic reticulum membrane similar to the effects of SRβ in rat and saccharomyces. In the present study, most tissues examined such as: brain, liver, lung, breast, colon, stomach, esophagus and testis showed a little nonhomogeneous expression of APMCF1. As a matter of fact, protein translocation across and insertion into membranes in cells are essential to all life forms, which might elucidate the results of a wide range expression pattern of APMCF1 in different normal human tissues.

On the other hand, in our preliminary study, APMCF1 was cloned as a novel apoptosis related gene whose transcripts were up regulated in apoptotic breast carcinoma MCF-7 cells and protein level was elevated in colon carcinoma [2,3]. Furthermore, ectogenic expression of APMCF1 could induce inhibition of HHCC growth. Results of cell cycle gene chips analysis showed up-regulation of p21 expression and down-regulation of TIMP3 in HHCC cells expressing ectogenic APMCF1, indicating that APMCF1 participates at least partially in cell cycle regulation through regulating genes such as p21 and TIMP3 [4]. The IHC study reported here showed its expression was up-regulated in the carcinoma tissues of liver, colon, esophagus, lung and breast carcinomas compared with their corresponding normal tissues, and the positive ratios of APMCF1 in liver, colon, esophagus, lung and breast carcinomas with a large samples were 96%, 80%, 57%, 58% and 34% respectively. These results together suggested APMCF1 might have a relationship with the cell growth, apoptosis of tumor cells or oncogenesis.

A recent study in microarrays analysis from Andrew Berchuck showed differences in survival of advanced ovarian cancers were reflected by distinct patterns of gene expression. APMCF1 together with T-cell differentiation protein (MAL), diphosphoinositol polyphosphate phosphohydrolase type2 (NUDT4), plakophilin 4 (PKP4), and signal sequence receptor (SSR1) were the top five genes involved, which were highly up-regulated in short-term survivors compared with long-term survivors and early-stage cases of ovarian cancers [23]. Many of the genes that were critical components of the patterns that discriminated between long-term and short-term survivors are known to affect the virulence of the malignant phenotype. Such as the MAL protein, a component of the protein machinery for apical transport in epithelial polarized cells and a component of membrane rafts which are micro-domains that play a central role in signal transduction acting as a scaffold in which molecules of signal transduction pathways can interact [24,25], has been shown expressed in ovarian cancers, most notably clear cell and serous cancers [26]. Thus we presume APMCF1 might be a critical factor in ovarian cancers though its expression was absent in the 2 cases of malignant ovarian tissues we detected. The additional independent expression study of APMCF1 is needed with large sample of ovarian cancers.

From the view of molecular structure, thus, as closely related to small G-protein superfamily, APMCF1 deserves further investigation whether it also has the function of regulating cell proliferation, apoptosis, tumorigenesis and metastasis. The present study has established foundation for new insight into the possible biological function of APMCF1 in tumor development and may represent an appealing potential therapeutic target in some tumors with high expression pattern of APMCF1.

## Conclusion

Our studies revealed a cytoplastic expression pattern of APMCF1 and up-regulation in many epithelium tumors suggesting APMCF1 may have potential relationship with oncogenesis. The data presented should serve as a useful reference for further studies of APMCF1 in tumorigenesis and provide a potential anti-tumor target.

## Competing interests

The authors declare that they have no competing interests.

## Authors' contributions

Wei Yan, Qing Li, Feng Zhu and Ruian Wang designed and supervised the experiments. Wei Yan contributed to pathologic morphological diagnosis. Qinlong Li, Kainan Li and Wenyong Wang carried out plasmid construction and cell transfection. Yaqing Zhang, Weihuang Wang and Jihong Cui performed immunohistochemistry. Yaqing Zhang, Qinlong Li and Wei Yan performed the statistic analysis and drafted the manuscript. All authors have read and approved the final version of the manuscript.
